# Evaluation of Sustained Persulfate Oxidant Release for Remediating Trichloroethylene Contaminated Low Permeability Soil in the Phreatic Zone

**DOI:** 10.1021/acsenvironau.4c00097

**Published:** 2025-01-30

**Authors:** Justine Kei T. Lim-Ortega, Chenju Liang, Analiza P. Rollon, Mark Daniel G. De Luna

**Affiliations:** 1 Environmental Engineering Program, National Graduate School of Engineering, University of Philippines Diliman, Quezon City 1101, Philippines; 2 Department of Environmental Engineering, National Chung Hsing University, 145 Xingda Rd., South Dist., Taichung 402, Taiwan; 3 Department of Chemical Engineering, University of Philippines Diliman, Quezon City 1101, Philippines

**Keywords:** contaminant rebound, controlled release, groundwater contamination, in situ chemical oxidation (ISCO), long-term remediation

## Abstract

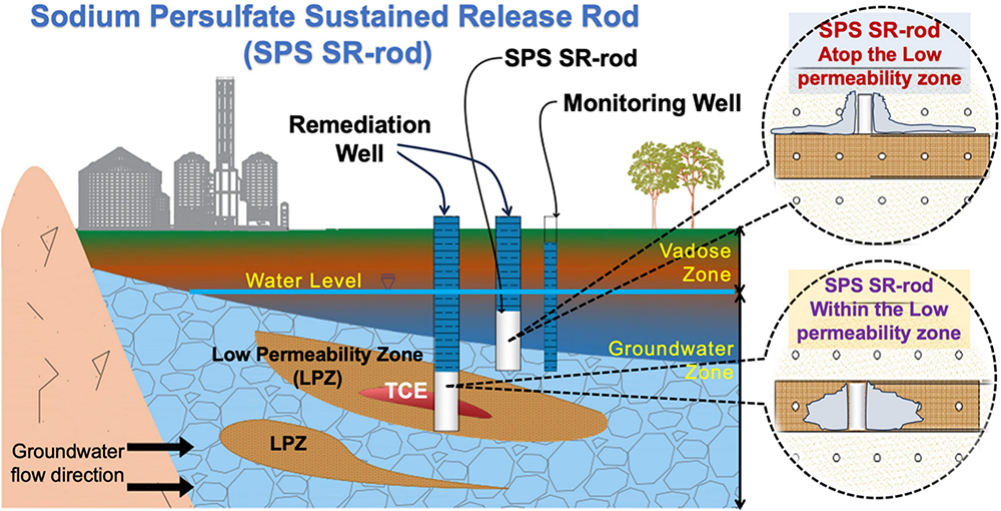

The back diffusion of trichloroethylene (TCE) between low permeability zones (LPZ) and transmissive zones in the subsurface presents remediation challenges. This study investigates in situ chemical oxidation (ISCO) using a sodium persulfate sustained release rod (SPS SR-rod) for potential TCE remediation in the LPZ within a two-dimensional sand tank. The tank simulates a dual permeability porous medium with hydraulic gradients of 0.01 and 0.05. The SPS SR-rod placed within the LPZ released an average PS concentration of ∼625 mg/L laterally, with initial peak concentrations of 7000–10,000 mg/L. When the rod was placed atop the LPZ, lower PS concentrations were observed compared to placement within the LPZ. A separate evaluation of both SPS SR-rod placements in a 2D sand tank injected with pure TCE tested the oxidant’s ability to address soil-sorbed TCE. The rod atop the LPZ can mitigate dual permeability layers and creates a depletion zone at the high permeability zone to reduce contaminant transport from the LPZ. The rod within the LPZ reduces TCE lateral dispersion. The persistence and slow release of SPS in the LPZ suggest that the SPS SR-rod could effectively extend the time period of ISCO remediation of low-concentration TCE in the LPZ and the surrounding environment.

## 1 Introduction

Industrialization has produced various synthetic chemicals for more than a century. One of the most used types of synthetic chemicals is chlorinated solvents, categorized as dense, nonaqueous phase liquids (DNAPLs). Chlorinated solvents in various industrial applications include degreasing agents, chemical intermediates, and dry-cleaning fluids.^1^ Discharging these types of wastes and their waste byproducts into the environment was prevalent due to the lack of environmental regulations during peak periods of DNAPL use, thus causing subsurface contamination, where they were released into the environment. Trichloroethylene (TCE) is the most frequently identified chlorinated solvent in the environment.^2^ Over the years, consistent detection and increased soil contamination have been associated with a growing list of identified TCE hazards to human health.^3−5^ While DNAPL in high permeability pathways and low moisture content regions depletes faster, DNAPL pooled above a capillary barrier can cause direct mass transfer into the low permeability zone (LPZ) via forward diffusion. Desorption and back diffusion of TCE DNAPL from the LPZ to the transmissive zone in the subsurface present a challenge for remediation. Mass removal by natural diffusion from LPZ is slow and, thus, contributes to the longevity of the contaminant in the subsurface.^6^

Generally, three classifications of soil and groundwater remediation methods have been applied on-site over the years: physical, chemical, and biological. Among these methods, in situ chemical oxidation (ISCO) is a commonly used technology due to its short remediation time and capacity to select different oxidants for different pollutants and geological conditions. ISCO using sulfate radicals (SO_4_^–^·), generated by thermal (e.g., 20–99 °C) persulfate (PS) (S_2_O_8_^2–^) activation shown in eq 1,^7,8^ can oxidize and mineralize TCE into nontoxic products such as carbon dioxide, following eq 2.^9^
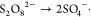
1
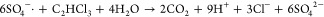
2

Hydrogeological conditions and characteristics at a contaminated site can affect the efficiency of these remediation methods. Dense soil can create difficulties in oxidant flow or diffusion, and high permeability zones (HPZs) can create preferential flows that bypass the LPZ. Soil with low permeability has lower water infiltration rates and a higher fluid-holding capacity. Low soil permeability reduces the span of horizontal dispersion of the organic contaminants, resulting in the containment of DNAPL in the LPZ until it diffuses back to the aquifer caused by changes including water content-associated capillary action, head pressure, concentration gradients, flow velocities, or temperature variation.^4,10^ The consequences of LPZ solvent contamination can be significant and long-lasting due to the transport behavior of low solubility DNAPL, which migrates slowly from micropore environmental compartments. This is due to several characteristics of the DNAPL solvent and the micropore environment including, but perhaps not limited to, density, surface tensions, absorption, adsorption, trapping, and low flow velocities. The longer retention time in the LPZ equates to potentially ten times longer remediation period for the traditional ISCO methods.^11^

Formulating controlled release materials (CRMs) in forms like oxidant gels, beads, pellets, tablets, and rods has been investigated.^5^ The development of sustained oxidant release technology aims to increase the treatment efficiency of the traditional ISCO method in LPZ. Table S1 (Supporting Information, SM) summarizes research studies on CRMs serving as long-term sources for continuous and stable supply of oxidants in remediation applications utilizing ISCO. Sustained release technology can overcome the problems associated with the traditional active oxidant flow and diffusion method. The CRMs have also been incorporated into different applications. For example, the pharmaceutical industry initially used the controlled release technology, where the drug is either physically coated or chemically bonded to create a sustained-release tablet, with a gradual drug release over time, minimizing the risk of overdose.^12^ Other applications of this technology include the supply of nutrients in agriculture, such as fertilizer application,^13,14^ food additives in the food industry,^15,16^ and in recent years, applications to soil and groundwater remediation.^5,8,17,18^

Liang and Chen (2017)^19^ pioneered the development of a sustained release rod utilizing paraffin wax and sodium persulfate (SPS), termed the SPS sustained release rod (SPS SR-rod). Their study described the initial release of SPS from the rod, confirmed through the reaction with a potassium iodide (KI) solution, resulting in a brown iodine (I_2_) indicator color in an aqueous setting. It was observed that a darker color solution was formed at the bottom of the flask due to the sinking of higher concentration, higher density SPS solution as SPS was released from the rod. Furthermore, based on the analysis of a matrix boundary diffusion-controlled two-film theory model, the results indicated the correlation between the radius of the rod and its minimum release time, providing a reference lifespan for field applications. Moreover, Liang and Weng (2022)^8^ evaluated the effectiveness of the rod in treating DNAPL-phase TCE. The SPS released from the rod degraded the aqueous TCE, dissolved from the TCE DNAPL droplets. Aqueous TCE was found to be less than 3 mg L^–1^ at more than 60 d of reaction time in the presence of the SPS SR-rod, while TCE dissolution reached approximately 700 mg L^–1^ in solution in the absence of the rod. The SPS SR-rod can create an oxidation zone via the diffusion mechanism by slowly releasing the oxidant to treat pollutants dissolved in the groundwater.

This study evaluated LPZ remediation through SPS SR-rod CRM technology to address the oxidant limiting approaches of the traditional ISCO methods by evaluating the long-term remediation of back diffusion of TCE contamination in the two-dimensional (2D) sand tank system. The first phase of this study aimed to investigate the sustained release of the PS concentration by determining its distribution contour in a saturated dual permeability porous media, at two different SPS SR-rod placements, atop and within the LPZ. The lateral distribution of PS concentration, persistence of the PS in the low permeability soil, release characteristics of PS in the target soil matrix over time, concentrations diffused into the low permeable soil, and residual PS remaining in the soil matrix were evaluated. The objective of the second phase of this study was to evaluate the potential remediation application of a sustained release ISCO technology to degrade residual TCE within the LPZ, using the SPS SR-rod in a 2D sand tank. The evaluation of sustained persulfate oxidant release in a 2D tank system offers valuable contributions to the understanding and potential application of this CRM ISCO remediation approach, especially for the commonly overlooked subsurface LPZ.

## 2 Methodology

### 2.1 Chemicals

Sodium persulfate (>99.0%, purchased from Evonik Active Oxygens, LLC.), a 0.02 M stock solution, was prepared to calibrate the benchtop visible spectrum spectrophotometer (Hach DR 3900 Spectrophotometer) for persulfate detection following the procedure developed by Liang et al.^20^ Potassium iodide (KI, > 99.5%) and sodium bicarbonate (NaHCO_3_, > 99.6%) were purchased from Union Chemical Works Ltd., Taiwan. Trichloroethylene (C_2_HCl_3_, 99.6% stabilized reagent) was purchased from Acros Organics. Soil was collected from a depth of approximately 2 m below the ground surface from farmland in southern Taiwan, referring to Table S2 (SM) for soil properties. The SPS SR-rod used in this study was manufactured according to the procedure by Liang and Chen.^19^

### 2.2 Experimental Design

**Figure 1:**
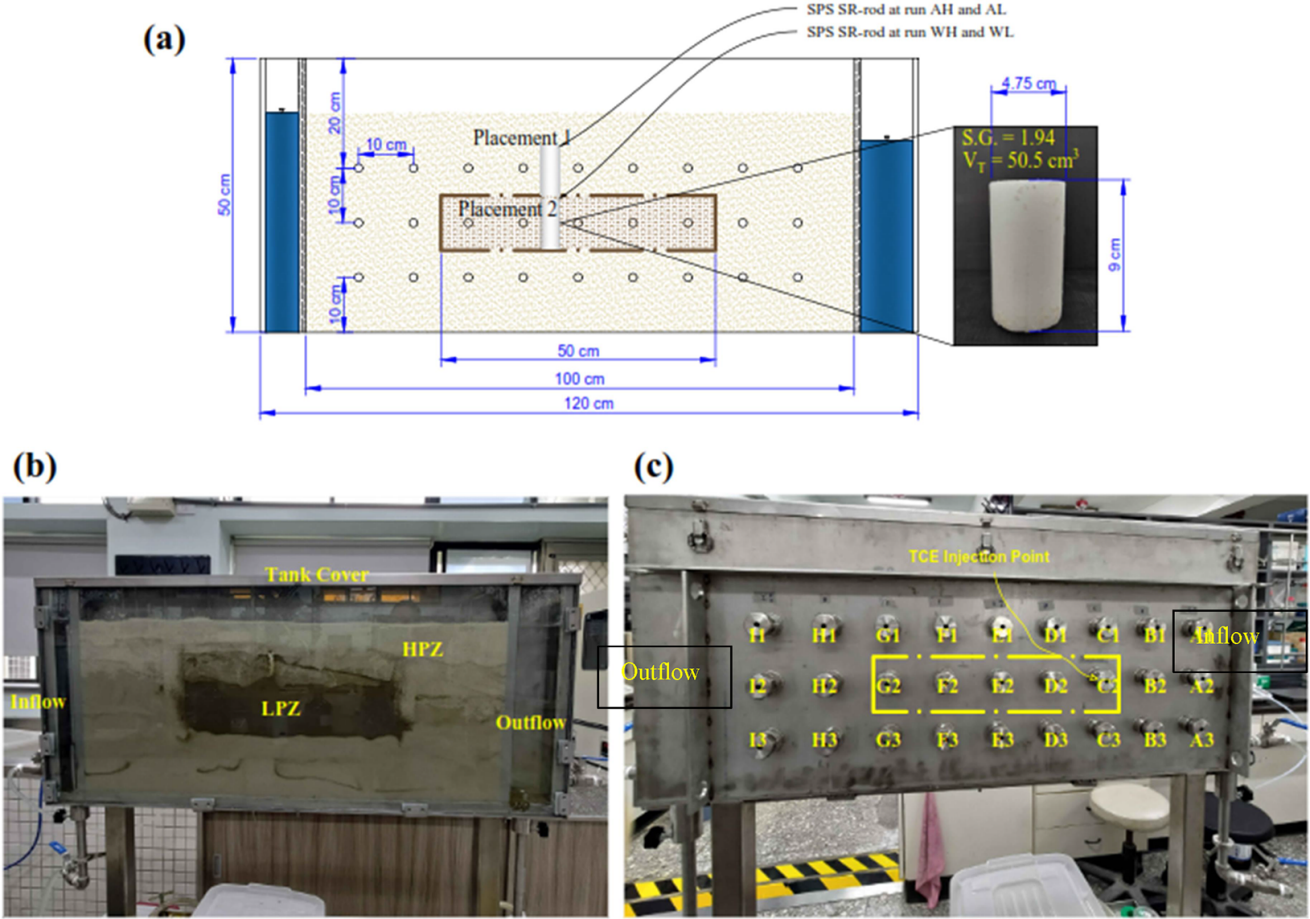
(a) Schematic diagram of the 2D sand tank showing the placements of the SPS SR-rod, water level, and sampling points. (b) Photograph of the front side of the 2D sand tank. (c) Photograph of the back side of the 2D sand tank.

The fabricated 2D sand tank represented a subsurface saturated condition and included low permeability strata. The study used two sets of tanks made of different materials. The first tank was made with acrylic sheets and was used to determine the concentration contour of persulfate, and the second tank was made of stainless steel and was used for TCE degradation experiments. Covering the stainless steel tank prevented TCE loss via vaporization and ensured the mass preservation between the vapor and dissolved phase TCE. Both tanks had identical design, dimensions, and arrangement of dual porous media, as shown in a schematic illustration in Figure 1(a). The two outer chambers, the inflow and the outflow, consisting of movable outflow pipes essential in adjusting the water levels, were used to create two different hydraulic gradients (i) at 0.05 and 0.01 with distinct specifications summarized in Table S3 (SM). The main chamber has 27 sampling ports to monitor the concentrations within the LPZ and its surrounding HPZ environment. Photographs of the front and back sides of the 2D stainless steel tank are shown in Figure 1(b),(c), respectively. The low permeability silty soil passing mesh number 200 was hand packed in the middle of the tank to represent the LPZ, while silica sand compliant to Ottawa standard (ASTM C778) with sieve size passing mesh number 30 and stopped at mesh number 50 filled the region surrounding the LPZ.^4^ The tank was filled with water and then slowly filled with porous media to obtain a saturated condition for the experiment. The SPS SR-rod was placed within the silty soil depth and atop it, before continuing to fill with silica sand. The experimental design flowchart is illustrated in Figure S1 (SM).

#### Phase I Experiments: Sustained PS Releases from the SPS SR-Rod within the 2D Sand Tank

The distribution contours of PS concentration were analyzed through two distinct rod placements: one directly delivering SPS within the LPZ and another positioned atop the LPZ. These experiments were conducted in a sand tank under two hydraulic gradients (i): 0.01 (low) and 0.05 (high); therefore, SPS SR-rod placement locations were denoted as WL (within low), AL (atop low), WH (within high), and AH (atop high), respectively. Both setups aim to mimic persulfate diffusion into the LPZ for long-term remediation. Each experimental setup was run for 30 d. The total volume of aqueous samples from 27 sampling ports was equivalent to 1% of the pore volume. Samples were taken by using a 3 mL syringe at an interval of 24 h. Sampling points C2, D2, E2, F2, and G2, shown in Figure 1, were located at the LPZ.

#### Phase II Experiments: Remediation Using the SPS SR-Rod to Degrade TCE within the 2D Sand Tank

A control experiment in the same soil stratification was injected with 200 μL of pure TCE at the C2 sampling port within the LPZ using a Hamilton gastight syringe (model 1725). After the injection of TCE, the water flow was continuously operated to enhance TCE distribution and dissolution in the aquifer, allowing it to reach sorption/dissolution equilibrium within the porous media.^21,22^ Aqueous samples were taken from four sampling ports (D2, E2, F2, and G2) within the LPZ, using a Hamilton gastight Syringe (model 710). In addition, samples from ports A2, E1, E3, I1, and I3 and the outflow were also analyzed for TCE, to evaluate dispersal. In the TCE degradation experiments, the preparation of sand tanks, where the rod was placed at WL and AL, followed the same procedure described above for the Phase I experiments.

### 2.3 Analysis

pH and ORP were measured using a benchtop pH/ISE/mV meter (HANNA HI5222) with a Mettler Toledo LE407 and Mettler Toledo InlabRedox, respectively. The water was prepared with an ELGA Micra Pure Water System. The benchtop spectrophotometer (Hach DR 3900 Spectrophotometer) was operated at a wavelength of 400 nm to determine PS concentration.^20^ For TCE with concentrations higher than 0.1 mg L^–1^, a gas chromatograph (GC, Agilent 7890B) equipped with a flame ionization detector (FID) and an OI Analytical 4660 Eclipse Purge-and-Trap sample concentrator were used. The GC was operated with a N_2_ carrier gas at a constant flow rate of 8.4 mL min^–1^, H_2_ flame gas at 40 mL min^–1^, an air supply at 400 mL min^–1^, N_2_ makeup gas flowing at 45 mL min^–1^, and a constant oven temperature at 40 °C.

TCE aqueous samples at trace concentration levels lower than 0.1 mg L^–1^ were analyzed using gas chromatography–mass spectrometry (GC-MS) on an Agilent 7890A GC, Agilent 5975C TAD Series MSD system equipped with an OI Analytical 4760 Eclipse Purge-and-Trap sample concentrator, and an OI Analytical 4551A Purge-and-Trap Water Autosampler. The GC-MS was operated using the parameters listed in Table S4 (SM). The data from the experiments were graphed using the software Origin Pro 2016.

## 3 Results and Discussion

### 3.1 Effect of SPS SR-Rod Placement on SPS Release

#### SPS SR-Rod within the LPZ

**Figure 2:**
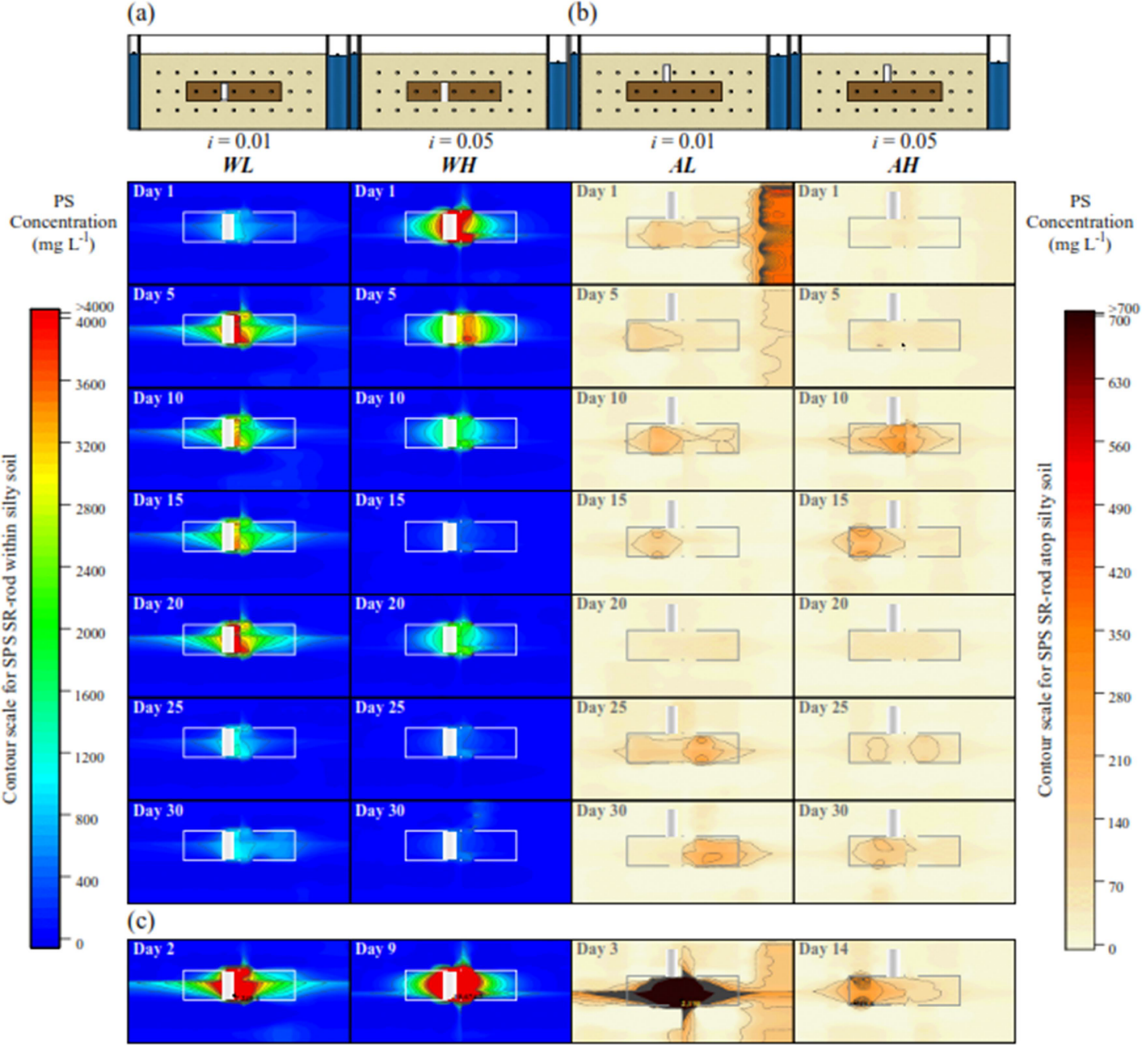
Concentration contours of persulfate released from the SPS SR-rod with time in dual permeability media in experimental conditions: (a) Placement of the SPS SR-rod within the LPZ, (b) placement of the SPS SR-rod atop the LPZ, and (c) determined peak concentration of persulfate with time.

In this test group where the SPS SR-rod was placed directly within the LPZ, between ports D2 and E2, at two hydraulic gradients, WL and WH released an average 625 mg L^–1^ PS concentration contour from at least 10 to 15 cm lateral distance from the rod within the LPZ, extending to approximately an additional 10 cm at the HPZ. The average PS value released from the SPS SR-rod was determined by averaging the results of the analysis from all sampling points of the individual phase 1 experiment concentration contour measurement results detailed in Figure 2. Aquired data associated with measured PS concentrations as a function of time can be seen in Figures S2 (run WH) and S3 (run WL) (SM). Comparing the concentrations above and below the LPZ, relatively higher measured PS concentrations were observed in the area below the LPZ (see Figures S2(a) vs S2(c) or Figures S3(a) vs S3(c)(SM)). This higher downgradient PS distribution is a result of the influence of PS solution density on its diffusion,^8,19^ where the density of PS solution increases with increasing concentration following eq 3, and is greater than that of water.^19^ The peak measured PS concentration in the LPZ was equivalent to 10,674 mg L^–1^ on day 9 (see Figure 2(c) and Figure S2(b) (SM)) for run WH and 7,240 mg L^–1^ on day 2 for run WL (see Figure 2(c) and Figure S3(b) (SM)).
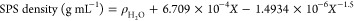
3where *X* is the SPS solution concentration (g L^–1^) and ρ_H_2_O_ is 0.99707 g mL^–1^ at 25 °C

Moreover, the PS concentration contour in Figure 2(a) shows that higher PS concentration levels were generally observed within the initial 5 day period, indicating that PS on the surface of the SPS SR-rod rapidly dissolved from the matrix phase and was released into the water phase, increasing the initial PS concentration.^19^ Subsequently, the concentration of PS released from the SPS SR-rod gradually decreased and stabilized in a later stage. This is because in the slow-release system, the oxidant release mechanism inside the oxidation rod is mainly determined by the concentration gradient between the PS concentration inside the rod and the outside environment. This resulted in approximately 800 to 1000 mg L^–1^ at run WH and 1000 to 6000 mg L^–1^ at run WL, with PS persisting in the LPZ for 30 d.

#### SPS SR-Rod atop the LPZ

In the subsequent experiments, the SPS SR-rod was placed atop the LPZ to observe the time-varying concentration distribution of PS in the 2D sand tank, caused by the density flow from the PS released at the top of LPZ. The SPS SR-rod placed atop the LPZ showed that the density driven migration of PS released sank downward, diffusing into the LPZ (as shown in Figure 2(b). PS release from the rod was evident as PS concentrations were continuously detected at an average value of ∼57 and ∼140 mg L^–1^ for AH and AL, respectively, as shown by the data in Figures S4 and S5 (SM)). The peak measured PS concentration in the LPZ was equivalent to 342 mg L^–1^ on day 14 for run AH (see Figure 2(c) and Figure S4(b) (SM)), and 1920 mg L^–1^ on day 3 for run AL (see Figure 2(c) and Figure S5(b) (SM)), respectively. This data show that the higher density of PS compared to that of water resulted in density and concentration gradients moving the PS through the LPZ, effectively explaining the higher concentration range of PS within the LPZ compared to the HPZ.

This SPS SR-rod placement aimed to continuously mitigate contaminant spread from the LPZ layered sites in the subsurface and establish a contamination depletion zone in those areas. Moreover, the purpose of this zone is to mitigate contaminant emissions from the LPZ to the extent that is feasible. During the treatment phase, if a dissolved source is present in a lower permeability soil, the diffusion of contaminants out of the LPZ can be reduced or eliminated by reducing the contaminant concentration at the interface.^23,24^ This reduction in the contaminant concentration creates a steeper concentration gradient at the interface, resulting in increased contaminant diffusive flux from the LPZ. This effect is temporary and will persist during active oxidative treatment and maintenance.

Under a low hydraulic gradient, the hydraulic retention time increased, dilution by flow was reduced, and the PS concentrations from the SPS SR-rod were higher. A high hydraulic gradient caused greater dilution of PS released, resulting in lower surrounding PS concentration contours. Greater variability in PS concentration differences was observed at the HPZ due to the rapid dissolution and dilution of PS released (see PS contours under AH in Figure 2(b)). A stable release was achieved, resulting in a high-concentration PS solution primarily distributed in the middle layer of the sand tank. The relatively lower concentration of PS in the upper and lower layers of the 2D sand tank resulted from the combined effects of the concentration gradient and the solute transport mechanism.

The placement of the SPS SR-rod within the LPZ significantly influences the oxidant release mechanism. When situated within the LPZ, the process is primarily governed by the concentration gradient as the minimal flow in this zone results in persulfate release, predominantly driven by concentration differences within the surrounding media. Conversely, positioning the SPS SR-rod atop the LPZ shifts the dynamics to a hydraulic gradient-controlled zone. In this case, the release of persulfate occurs through a dissolution-diffusion process, where the interaction with the overlying water leads to dilution and a reduced local persulfate concentration. This setup facilitates a dual migration pattern: lateral migration driven by water flow and downward movement due to density differences between the persulfate solution and water. The interplay of these movements contributes to a more complex distribution, resulting in lower detected concentrations of persulfate in the surrounding environment.

**Figure 3:**
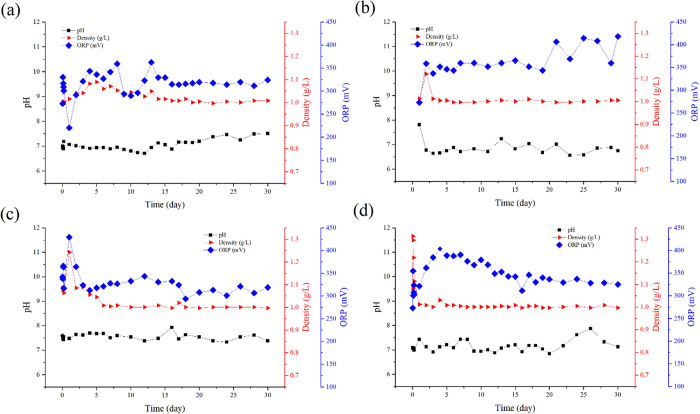
pH, density, and ORP observed in the outflow of the tank under the experimental conditions (a) WL, (b) WH, (c) AL, and (d) AH.

During PS release from the rod, changes occurred in the system pH (Figure 3). From an approximate neutral pH of the injected water, pH slightly declined to a range of 6.5. Its inverse relationship with the ORP shows an increased value from 280 to 350 mV. The ORP is lower in conditions WL (Figure 3(a)) and AL (Figure 3(c)) due to the slower dispersion rate at the lower flow. Substantially elevated ORP and substantially depressed pH levels all point to creating a strongly oxidizing environment. The activation of PS generates a range of radicals that may aid in the degradation of TCE and enhance its oxidation potential. Depending on the solution’s pH, these various radicals take prominence: in an acidic solution, SO_4_^–^· is the primary radical; in a neutral solution, both hydroxyl radicals (·OH) and SO_4_^–^· are equally active; in a basic solution, ·OH dominates. These various radicals offer distinct advantages in the degradation of TCE, contributing to its effective treatment.^25^ The mass of SPS released by measuring the changes of weight in the SPS SR-rods before and after 1 month use in the experiment measured approximately 6–10% and 3–5% of SPS released for *i* = 0.05 and *i* = 0.01, respectively (Table S5 (SM)). At 0.01 hydraulic gradient, the PS mass released from the rod is roughly half of the mass released when the hydraulic gradient is 0.05, regardless of the placement in the LPZ. Residual PS concentration levels were higher when the hydraulic gradient was 0.01 (Figure 2). The persistence of released PS outside the rod affects the release rate, leading to a lower diffusion rate at reduced concentration gradients between the interior and exterior of the rod.^19^

According to Liang et al.,^19^ who developed and characterized the SPS SR-rod, the SPS SR-rod matrix diffusion control systems uniformly disperse or dissolve the core material in the macromolecular polymer (matrix) and use the concentration gradient inside and outside as the driving force to diffuse the core material into the external environment. The matrix diffusion control system is where the core material is dispersed uniformly in the matrix and the outermost layer is in direct contact with the external environment. Therefore, the persistence of persulfate in the environment slows diffusion rather than limits it. It is noted that persulfate is known to exhibit slow to moderate reactions with soil organic matter, which can contribute to a reduction in the oxidant concentration within the system. However, the consistent levels of ORP observed over time indicate the sustained presence of an oxidant in the system, consistent with the slow-release mechanism. This mechanism may reduce the scavenging of radicals, thereby enhancing the degradation of TCE.^26^ The variations in PS concentration in the outflow under Phase I experimental conditions (WL, WH, AL, and AH) are illustrated in Figure S6 (SM). The data reveal a consistent trend across all conditions, characterized by an initial rapid increase in PS concentration followed by stabilization at relatively low levels.

### 3.2 Effects of SPS SR-Rod Placement on TCE Degradation

#### TCE Concentration Determination without the SPS SR-Rod

**Figure 4:**
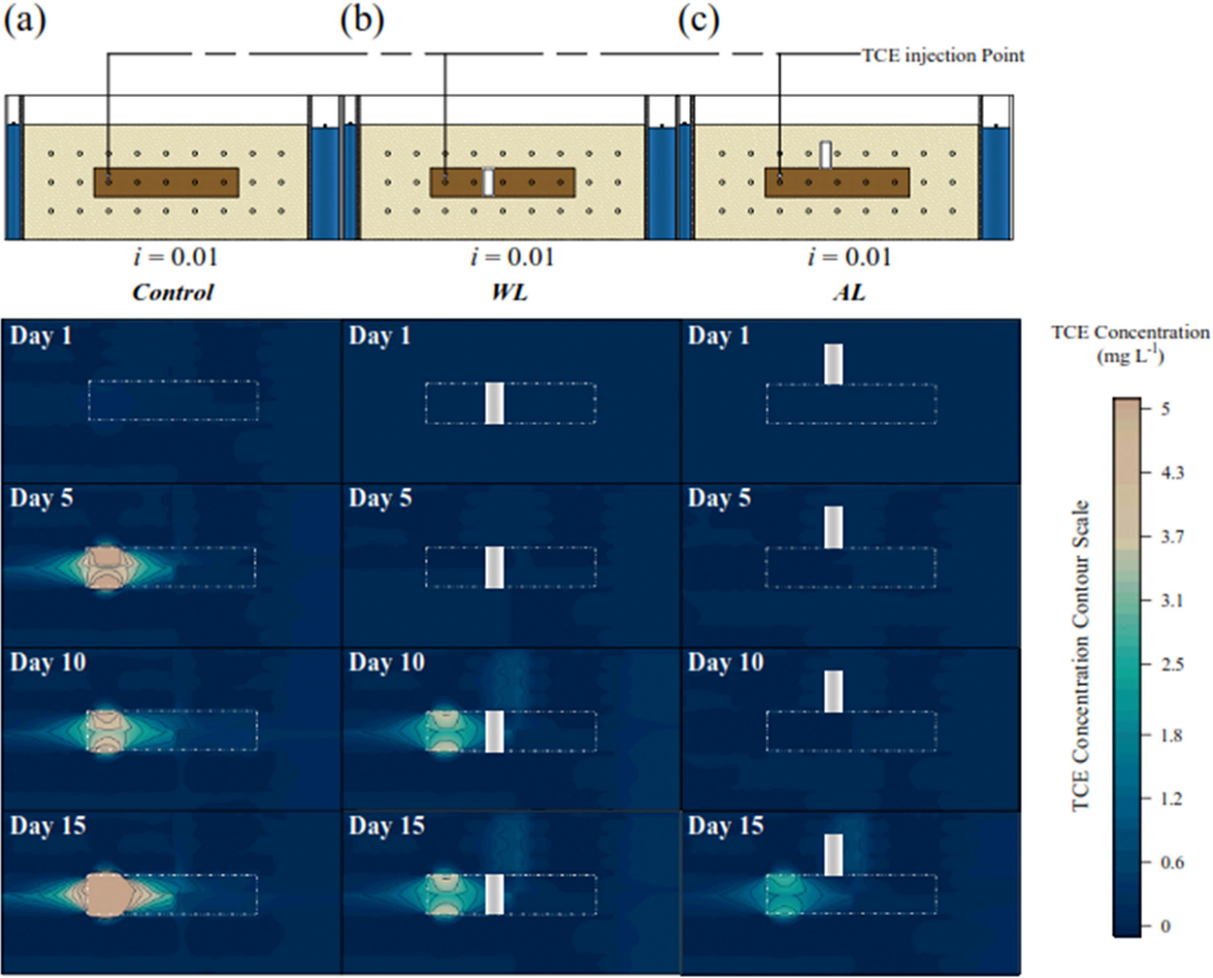
Concentration contour of TCE with time in saturated conditions in dual permeability media in experimental conditions (a) without SPS SR-rod. (b) Placement of the SPS SR-rod within the LPZ and (c) placement of the SPS SR-rod atop the LPZ.

A control experiment was conducted for the determination of TCE concentration distribution when residual TCE was present in the LPZ, without placement of the SPS SR-rod, under the low hydraulic conductivity condition (*i* = 0.01). After injection of 200 μL of TCE at port C2, the concentration was monitored (data presented in Figure S7 (SM)), and subsequently there was no TCE detection at the HPZ sampling points until the fifth day of the run, as shown in the TCE contour diagram in Figure 3(a). TCE sorption onto soil particles and desorption from soil back into the aqueous phase would occur before the observation of TCE on the fifth day. The sorption of TCE onto the soil is a phenomenon called hydrophobic partitioning at the uncharged region of soil organic matter and adsorption pore filling. These mechanisms follow the van der Waals forces between hydrophobic chemicals that exhibit reduced interactions with polar water molecules, leading to decreased entropy.^27^ On the other hand, transporting the substance to the soil sorption site is related to heterogeneous flow or intrasorbent diffusion, limiting the interaction between sorbate and sorbent.^28−30^ This was also observed on day 10 (Figure 4(a)), wherein the TCE contour showed lower concentrations than the results from day five and the highest concentration on day 15. During the 15 day duration, zero to minimal TCE concentrations were observed at the HPZ. However, relatively high TCE concentrations (e.g., 0.2–1.0 mg L^–1^) were mainly detected within the LPZ, and gradually increased in the outflow to greater than 0.05 mg L^–1^ (see Figure S7 (SM)). Following the injection of TCE, aliquot testing was conducted to monitor the concentration levels. By the 15th day, TCE concentrations exceeding 0.05 mg L^–1^ were consistently detected at a 200 μL injection volume across all LPZ sampling ports and the outflow in the control test. With the application of the SPS SR-rod, TCE degradation by oxidation over the 15 day period can be assessed in comparison to the control test, with an anticipated reduction in the TCE concentration in the aqueous solution anticipated. This expectation is supported by Phase 1 results, which demonstrated that PS release from the rod stabilized after an initial rapid release within the first 5 days, remaining effective in degrading TCE. Consequently, a 15 day experimental period was established for Phase 2 to evaluate the impact of SPS SR-rod placement on TCE degradation.

#### TCE Degradation with SPS SR-Rod Placed within LPZ

TCE concentrations were measured in the LPZ immediately following placement of the SPS SR-rod within the LPZ (illustrated in Figure 4(b)). No TCE concentration was detected until the 10th day. This result could be attributed to TCE’s initial reaction with the persulfate released to the LPZ and its surrounding environment. The PS concentration determined on the 10th day was approximately 77% lower than that of the Phase I WL PS release experiment. Furthermore, the GC-MS detections for the TCE degradation experiment measured the presence of cis-1,2-dichloroethene (cis-1,2-DCE), but only at a concentration of 3 × 10^–6^ mg L^–1^, which is below the MS quantification limit of 0.17 μg L^–1^ for cis-1,2-DCE. The result signifies that cis-1,2-DCE is only short-lived in the LPZ strata. Moreover, because DCE and VC are significantly more prone to oxidation and destruction, they are rarely measured in groundwater during active ISCO PS application.^31^

From day 10 to day 15, there was an approximately 0.5 mg L^–1^ TCE concentration at point E1, the sampling point in HPZ positioned slightly above the placement of the SPS SR-rod in the LPZ. In this case, the oxidant rod acted as a barrier with increased velocity from the release of SPS from the rod, causing upward flow instead of lateral dispersion. This phenomenon resulted in TCE at E1, D2, and outflow at levels above the TCE groundwater limit concentration (0.05 mg/L) until the 15th day of the run, while no TCE concentrations were observed at the other sampling ports (see Figure S8 (SM)).

#### TCE Degradation with SPS SR-Rod Placed atop LPZ

During run AL, TCE concentrations were detected in the outflow on the 15th day, and this condition was comparable to that observed in run WL on the fifth day (see Figure S9 (SM)). That is because the PS released under the AL condition degraded the diffused TCE that migrated to the HPZ. On the 15th day, as the TCE continued to disperse in the LPZ, its concentration increased (see Figure 4(c)), causing fewer sorption sites to become available and decreasing soil-water partitioning.^32^ No TCE concentrations were observed at the HPZ (Figure S9 (SM)). The TCE magnitude and distance between the source and the soil permeability interface drive diffusive processes in lower permeability soils. The mass flux decreases as TCE travels further, resulting in lower aqueous concentrations. As the distance between the source and the permeable unit increases, the concentration of TCE in the flowing groundwater decreases.^32^ The released PS present above the LPZ acted as an oxidative barrier to destroy the dissolved TCE leaving the LPZ. The PS that penetrated PS into the LPZ (see Figure 2(b) under the run AL) is also capable of oxidizing TCE, and no TCE was detected within the LPZ, except at the sampling port (D2), which is close to the C2 TCE injection port. The PS concentrations at the outlet of the tank under Phase 2 experimental conditions (WL and AL) are shown in Figure S10 (SM) for reference.

## 4 Conclusions

The persistence of persulfate in the LPZ and its slow release in the subsurface support that the SPS SR-rod may be an efficiently controlled release material and can extend the ISCO remediation of TCE in low-concentration scenarios, in and around the LPZ environment. With SPS SR-rod application, challenges of soil-sorbed TCE, such as back diffusion, the difficulty of treatment in LPZs due to preferential pathways, and tailing and rebound, are addressed. Placements of the SPS SR-rods within and atop the LPZs provide significant efficiency enhancements in their application to on-site remediation. The SPS SR-rod placed atop the LPZ continuously mitigates the LPZ layered sites in the subsurface. It establishes a dense depletion zone at the HPZ to decrease contaminant emissions from the LPZ to lower levels. Also, with the SPS SR-rod placed within the LPZ, it readily contains the TCE in the area of concern, reducing lateral dispersion and reducing contamination of surrounding areas in the subsurface. A strategic combination of the two placements can increase the remediation efficiency. By continuously reducing released concentrations of TCE over an extended period, the technology ensures sustained treatment and degradation of TCE in low-concentration scenarios. This approach allows for efficient and effective remediation, while minimizing the hazard of adverse environmental effects or excessive chemical usage. This study helps plan a remediation design using the SPS SR-rod, especially in a geologic setting involving LPZ. The placement scenarios help plan the drilling of injection wells on site. The application of the SPS SR-rod can extend the remediation time period at TCE-contaminated sites, where back diffusion and tailing of TCE constitute significant concern. This study marks an initial effort to investigate such a scenario under controlled conditions by using the SPS SR-rod. Notably, although the TCE release behavior in this setup differs from aged contaminated soil, it provides a comparable representation of residual TCE concentrations, serving as a reference for real-world conditions. Future researchers on this topic may consider an experimental design that mimics the environmental scenario of residual TCE before applying the SPS SR-rod. Supplementing the study with additional investigations in more complex and realistic field settings could provide a more comprehensive understanding of the remediation potential. Such studies would allow for the effective assessment of long-term oxidation effects, including surface scaling, and evaluate the applicability of the sustained persulfate release approach for TCE-contaminated, low-permeability silty soils.

## Data Availability

Data will be made available on request.

## Supporting Information Available

Studies on TCE remediation by the ISCO method (Table S1); properties of soils used in this study (Table S2); specification of the 2D sand tank (Table S3); GC/MS operating conditions (Table S4); mass of SPS released in Phase I experiments (Table S5); experimental design flowchart (Figure S1); PS concentration variation as a function of operation time at run WH (Figure S2); PS concentration variation as a function of operation time at run WL (Figure S3); PS concentration variation as a function of operation time at run AH (Figure S4); PS concentration variation as a function of operation time at run AL (Figure S5); PS concentration in the outflow of the tank under Phase 1 experimental conditions (Figure S6); TCE concentration variation as a function of operation time without SPS SR-rod (Figure S7); TCE concentration variation as a function of operation time at run WL (Figure S8); TCE concentration variation as a function of operation time at run AL (Figure S9); PS concentration in the outflow of the tank under Phase 2 experimental conditions (Figure S10) (PDF)
