# Supplementary material for: Evaluation of Sustained Persulfate Oxidant Release for Remediating Trichloroethylene Contaminated Low Permeability Soil in the Phreatic Zone
Source: ACS Environ Au. 2025 Jan 30;5(2):211–9. doi: 10.1021/acsenvironau.4c00097 (PMC11926746; doi:10.1021/acsenvironau.4c00097)
Supplement: Supplementary file 1 — vg4c00097_si_001.pdf [file vg4c00097_si_001.pdf]

Supplementary Materials

Evaluation of sustained persulfate oxidant release for remediating trichloroethylene contaminated low permeability soil in the phreatic zone

Justine Kei T. Lim-Ortega [1], Chenju Liang [2*], Analiza P. Rollon [1,3], Mark Daniel G. de Luna[1,3]

[1] Environmental Engineering Program, National Graduate School of Engineering, University of Philippines Diliman, Quezon City 1101, Philippines

[2] Department of Environmental Engineering, National Chung Hsing University, 145 Xingda Rd., South Dist., Taichung 402, Taiwan

[3] Department of Chemical Engineering, University of Philippines Diliman, Quezon City 1101, Philippines

* Corresponding author: Email address: cliang@nchu.edu.tw; Tel: +886-4-22856610,

Table S1. Studies on TCE remediation by the ISCO method

Oxidant	Binding Agent	Morphology	Location	Testing period	Reference
$KMnO_4$	paraffin	candle	Field	5 years	[1]
$KMnO_4$	polymer resin	particles	Lab – tank	20 days	[2]
$KMnO_4$	paraffin – silica	gel	Lab –column	33 days	[3]
$KMnO_4$	polycaprolactone – Starch	candle	Lab – column	76 days	[4]
$KMnO_4$	polymer	particles	Lab – bottle	90 days	[5]
$KMnO_4$	fumed silica	gel	Lab – column	8 days	[6]
$KMnO_4$	stearic acid	particles	Lab – bottle	9 days	[7]
$S_2O_8^{2-}$	HPMC	tablets	Lab – bottle	12 days	[8]
$S_2O_8^{2-}$	zeolite, diatomite, silica	particles	Lab – bottle	10 days	[9]
$S_2O_8^{2-}$	paraffin	candle	Lab – tank	60 days	[10]
$S_2O_8^{2-}$	HPMC – biochar	particles	Lab – bottle	15 days	[11]

References:

[1] M. Christenson, A. Kambhu, J. Reece, S. Comfort, and L. Brunner, “A five-year performance review of field-scale, Slow-release permanganate candles with recommendations for second-generation improvements,” *Chemosphere*, vol. 150, pp. 239–247, May 2016, doi: 10.1016/j.chemosphere.2016.01.125.

[2] B. S. Lee *et al.*, “Efficacy of controlled-release KMnO4 (CRP) for controlling dissolved TCE plume in groundwater: A large flow-tank study,” *Chemosphere*, vol. 74, no. 6, pp. 745–750, Feb. 2009, doi: 10.1016/j.chemosphere.2008.10.062.

[3] E. S. Lee and N. Gupta, "Development and characterization of colloidal silica-based slow-release permanganate gel (SRP-G): Laboratory investigations," *Chemosphere*, vol. 109, pp. 195–201, 2014, doi: 10.1016/j.chemosphere.2014.01.020.

[4] S. H. Liang, K. F. Chen, C. S. Wu, Y. H. Lin, and C. M. Kao, "Development of KMnO4-releasing composites for in situ chemical oxidation of TCE-contaminated groundwater," *Water Res*, vol. 54, pp. 149–158, May 2014, doi: 10.1016/J.WATRES.2014.01.068.

[5] S. Luster-Teasley, P. Onochie, and V. Shirley, "Encapsulation of potassium permanganate oxidant in biodegradable polymers to develop a novel form of controlled-release remediation," *Emerging Environmental Technologies*, vol. 2, pp. 39–55, 2010, doi: 10.1007/978-90-481-3352-9_2/COVER.

[6] S. Yang, M. Oostrom, M. J. Truex, G. Li, and L. Zhong, "Injectable silica–permanganate gel as a slow-release MnO4− source for groundwater remediation: rheological properties and release dynamics," *Environ Sci Process Impacts*, vol. 18, no. 2, pp. 256–264, Feb. 2016, doi: 10.1039/C5EM00559K.

[7] B. Yuan, F. Li, Y. Chen, and M. L. Fu, "Laboratory-scale column study for remediation of TCE-contaminated aquifers using three-section controlled-release potassium permanganate barriers," *J Environ Sci (China)*, vol. 25, no. 5, pp. 971–977, May 2013, doi: 10.1016/S1001-0742(12)60134-X.

[8] Y. C. Chang, K. F. Chen, T. Y. Chen, H. H. Chen, W. Y. Chen, and Y. C. Mao, "Development of novel persulfate tablets for passive trichloroethylene (TCE)-contaminated groundwater remediation," *Chemosphere*, vol. 295, p. 133906, May 2022, doi: 10.1016/J.CHEMOSPHERE.2022.133906.

[9] P. T. Pham *et al.*, “Sustained release of persulfate from inert inorganic materials for groundwater remediation,” *Chemosphere*, vol. 259, Nov. 2020, doi: 10.1016/j.chemosphere.2020.127508.

[10] C. Liang and C. Y. Weng, “Evaluation of alkaline activated sodium persulfate sustained release rod for the removal of dissolved trichloroethylene,” *J Hazard Mater*, vol. 439, Oct. 2022, doi: 10.1016/j.jhazmat.2022.129657.

[11] T. M. Nguyen, H. H. Chen, Y. C. Chang, T. C. Ning, and K. F. Chen, “Remediation of groundwater contaminated with trichloroethylene (TCE) using a long-lasting persulfate/biochar barrier,” *Chemosphere*, vol. 333, p. 138954, Aug. 2023, doi: 10.1016/J.CHEMOSPHERE.2023.138954.

Table S2. Properties of soils used in this study

Characteristics	Soil	Silica sand
Function	Low Permeability Zone	High Permeability Zone
Sand,%	76.1	Particles passing through a #30 sieve (0.59 mm) and retained on a #50 sieve (0.3 mm)
Clay, %	4.7	
Silt, %	19.2	
pH	8.0	6.9
Water content, %	0.80	n.d.
Total organic carbon (TOC), % by wt.	0. 157	0.022
Cation exchange capacity (CEC), meq/100g	6.10	n.d.
Fe, mg/kg	19069 ± 220	n.d.
Cu, mg/kg	49.6 ± 18.2	n.d.
Mn, mg/kg	296.7 ± 12.4	n.d.
Particle density, g/cm^3	2.40	2.65
Bulk density,	1.49	1.67
Porosity$^{(*)}$	0.38	0.37

Note:

n.d.: not determined.

Analysis of soil properties refers to the publication, Hou, Y., Liang, C.* 2022. Bisulfite reduction of soil iron for the reductive degradation of trichloroethylene. Chemosphere 286: 131818.

* When using the graduated cylinder method to measure soils with a significant silt/clay content, clay typically exhibits high porosity due to the negative charge on the surfaces of its book-shaped particles.

Table S3. Specification of 2D Sand Tank

Specification	Unit	Higher hydraulic gradient	Lower hydraulic gradient
Tank Dimensions	cm	100 L × 5 W × 50 H	
Total Volume	cm^3	25000	
Total Pore Volume (PV)	cm^3	6580	
Porosity		0.38	
Flow rate	$cm^3\ min^{-1}$	87.5	17.5
Hydraulic gradient		0.05	0.01
Inflow head	cm	40	40
Outflow head	cm	35	39
Discharge velocity	$cm\ s^{-1}$	3.65×10^{-4}	7.29×10^{-5}

Table S4. GC/MS Operating Conditions

	GC	MS	
Injector temperature	190	Data acquire mode	Scan and SIM
Injector volume	1 µL	Scan mass range	50-300 amu
Column flow	1 mL/min (He)	Scan speed	Normal
Oven temperature	190 ^{o}C	SIM ion	123, 77, 93
Split ratio	Splitless	Transfer line	250°C
LTM program	40°C (hold 2 min) to 190°C @15°C/min	Ion source	230°C
		Quad.	150°C
Column	HP-5	Solvent delay	1.66-5 min

Table S5. Mass of SPS released in Phase I experiments

	Hydraulic Gradient			
	***i* = 0.01**		***i* =0.05**	
**Description**	**WL**	**AL**	**WH**	**AH**
Initial weight, g	217.14	204.07	220.00	216.99
Final weight, g	207.23	197.80	199.04	204.48
SPS released, %	4.6	3.1	9.5	5.8

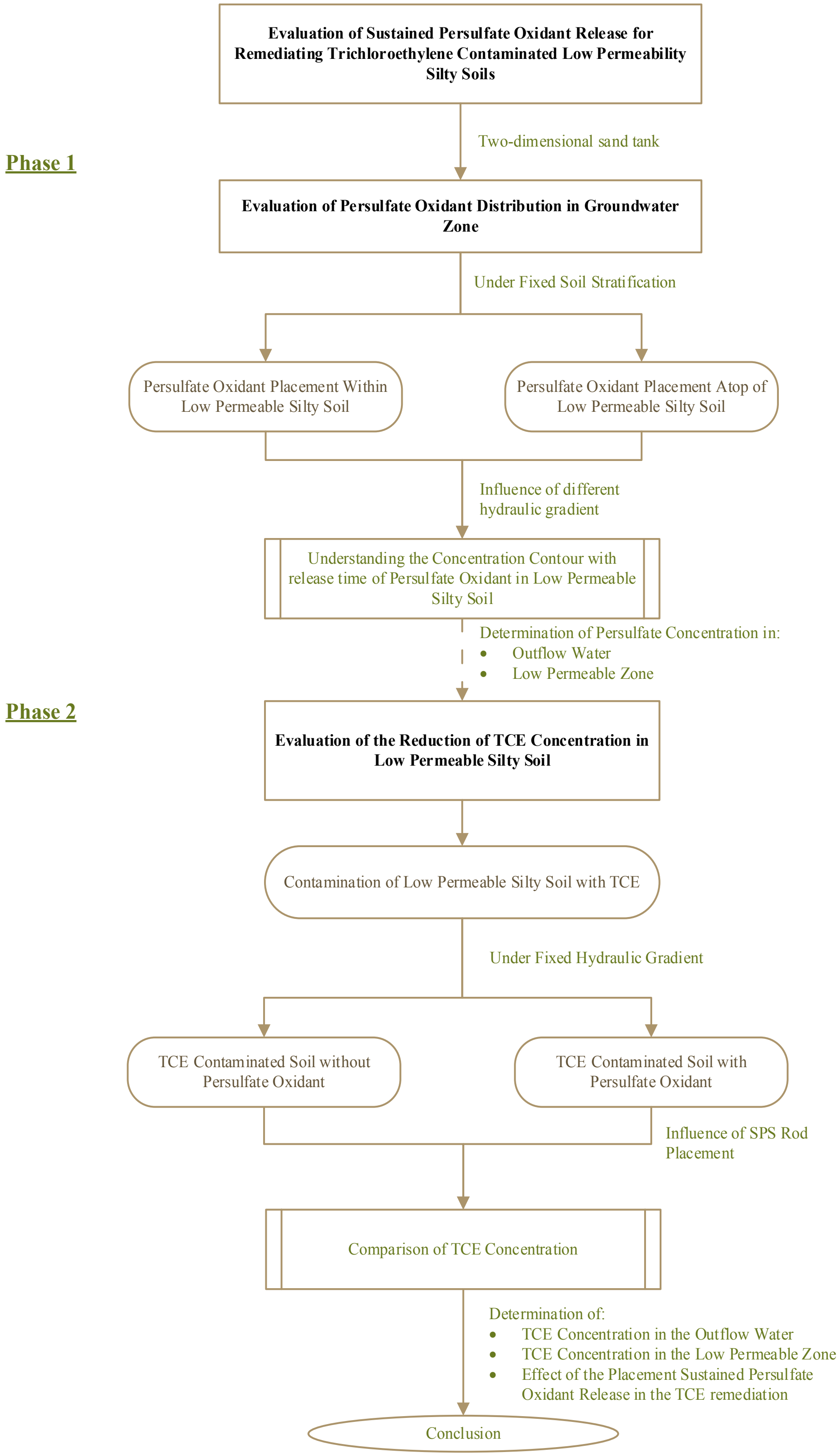

Figure S1. Experimental design flow chart

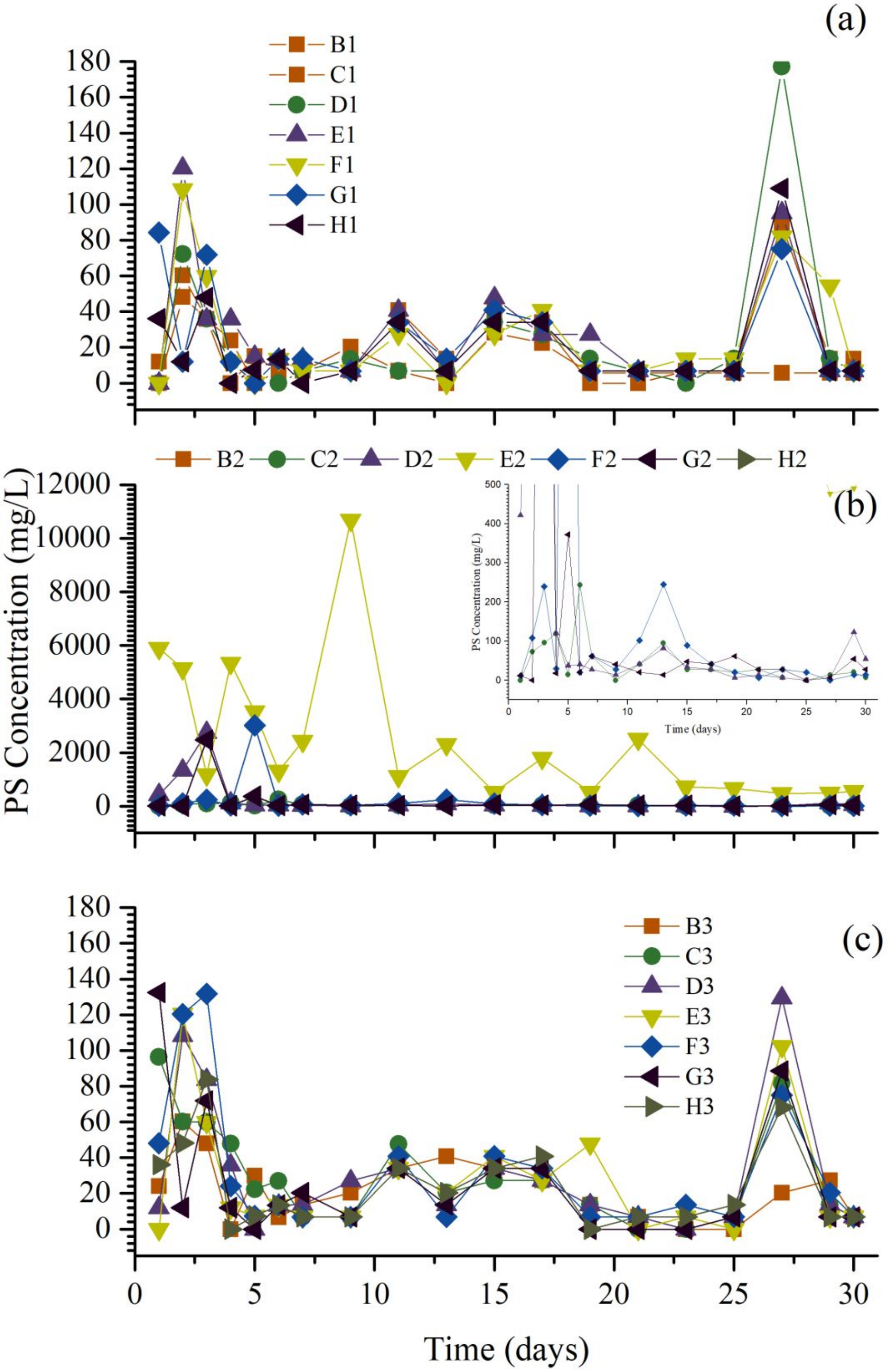

Figure S2. PS concentration variation as a function of operation time at run WH: (a) sampling port row 1, (b) sampling port in LPZ (row 2), and (c) sampling port row 3.

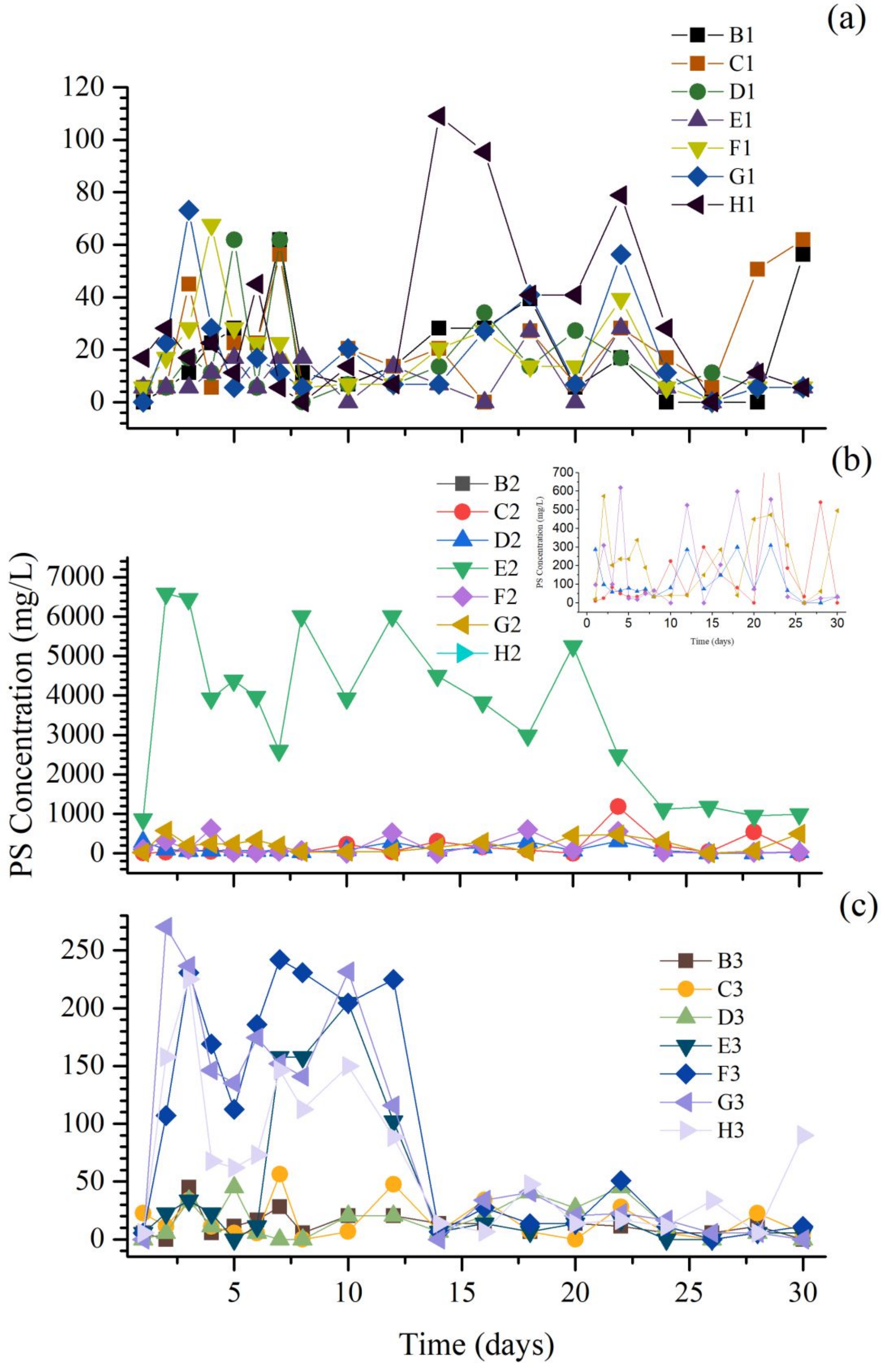

Figure S3. PS concentration variation as a function of operation time at run WL: (a) sampling port row 1, (b) sampling port in LPZ (row 2), and (c) sampling port row 3.

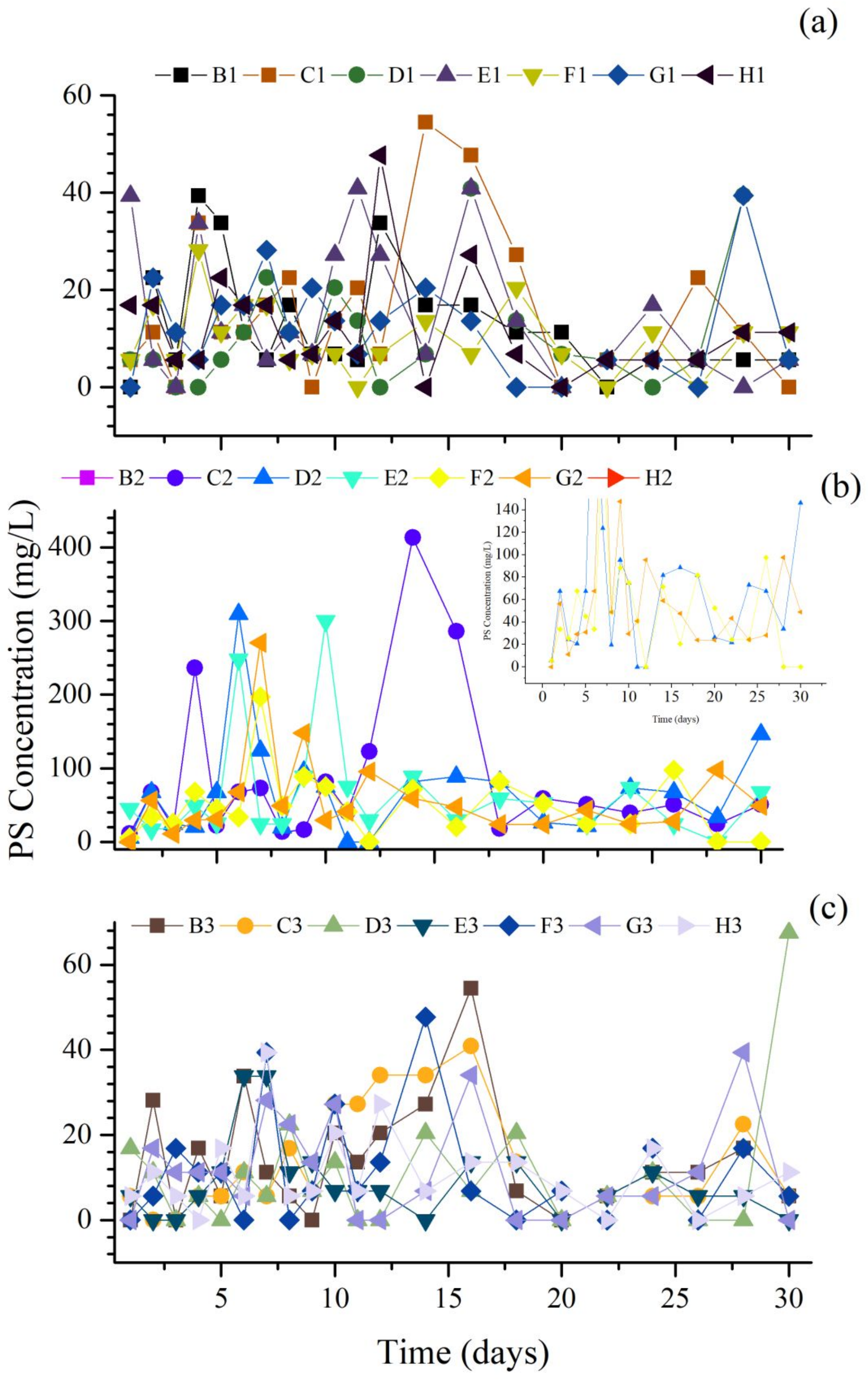

Figure S4. PS concentration variation as a function of operation time at run AH: (a) sampling point row 1, (b) sampling point in LPZ (row 2), and (c) sampling point row 3

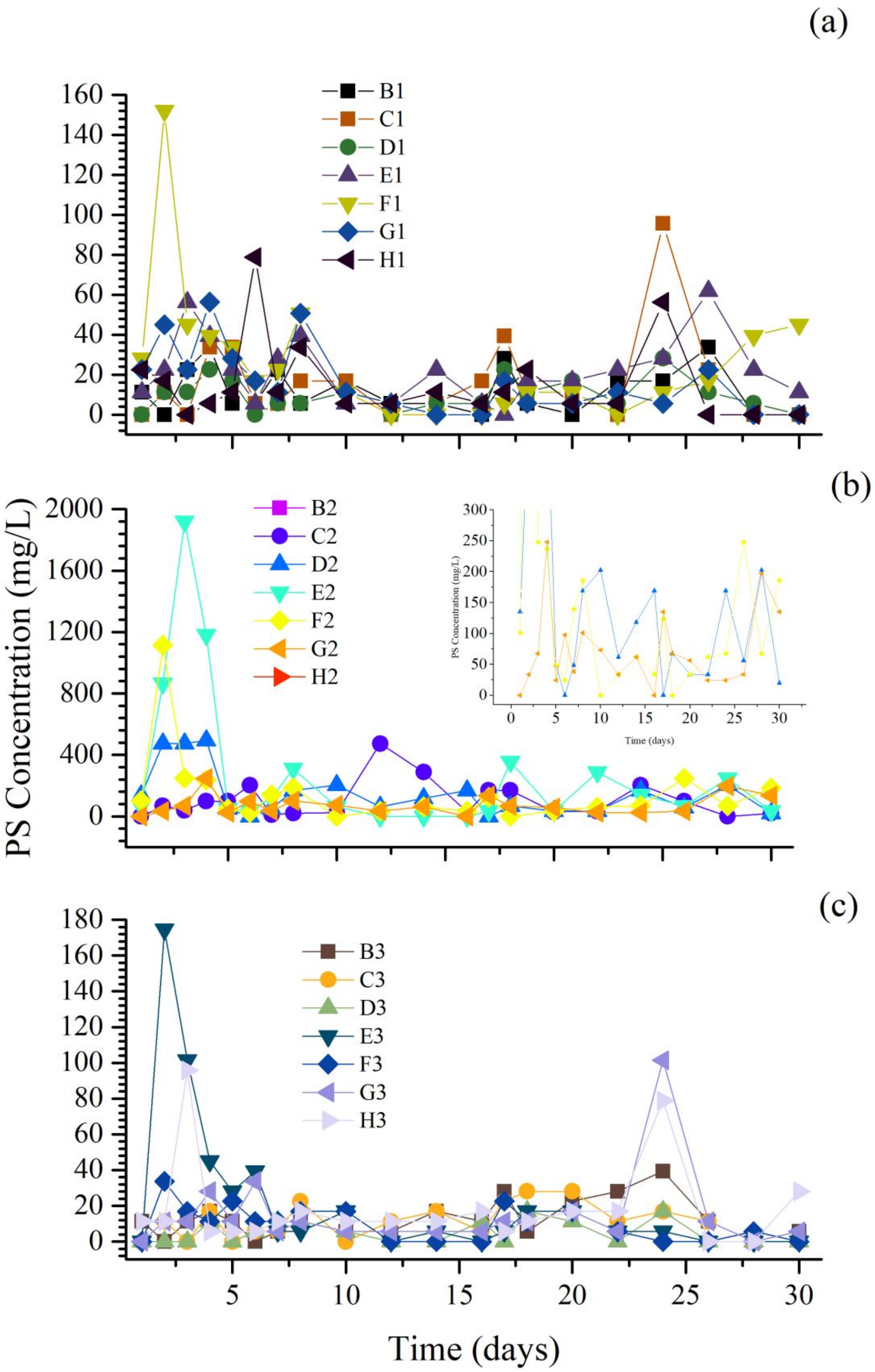

Figure S5. PS concentration variation as a function of operation time at run AL: (a) sampling point row 1, (b) sampling point in LPZ (row 2), (c) sampling point row 3.

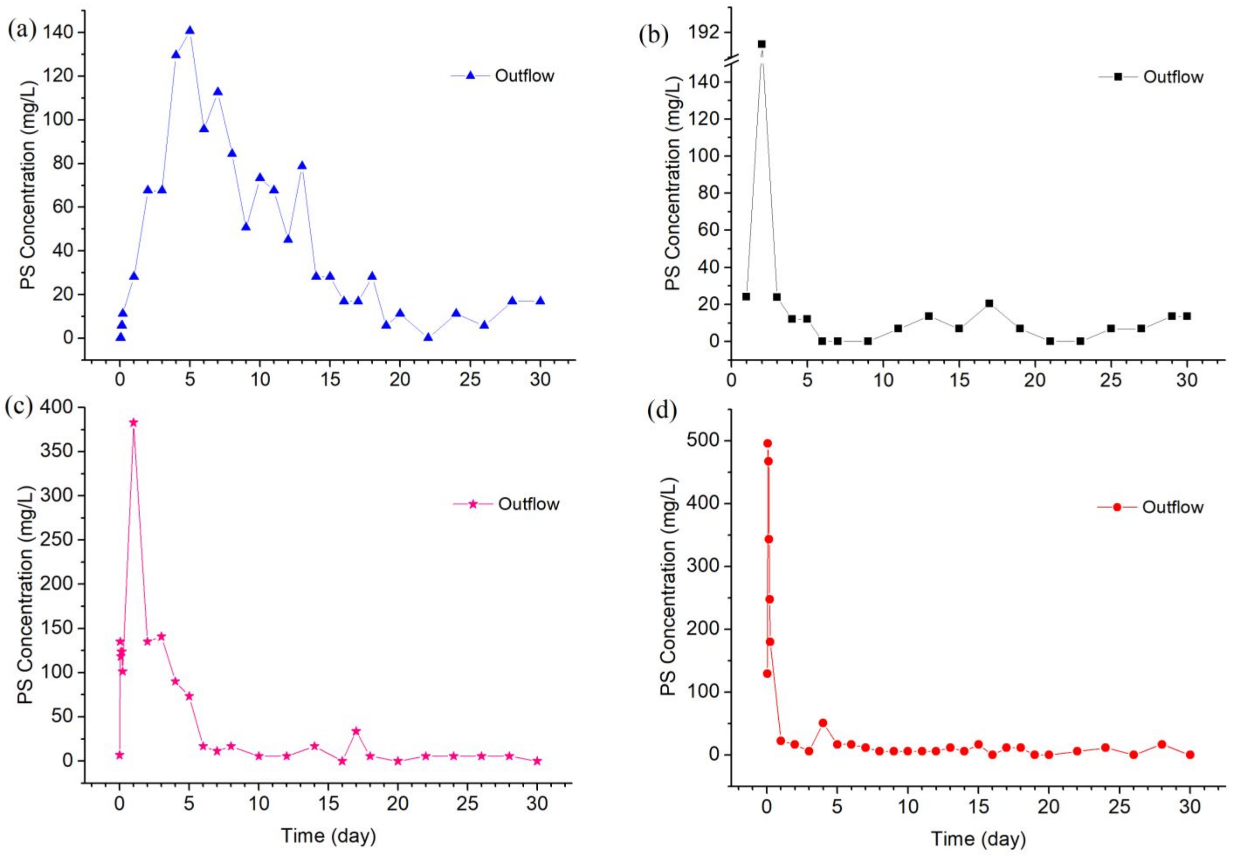

Figure S6. PS concentration in the outflow of the tank under Phase 1 experimental conditions (a) WL, (b) WH, (c) AL, and (d) AH.

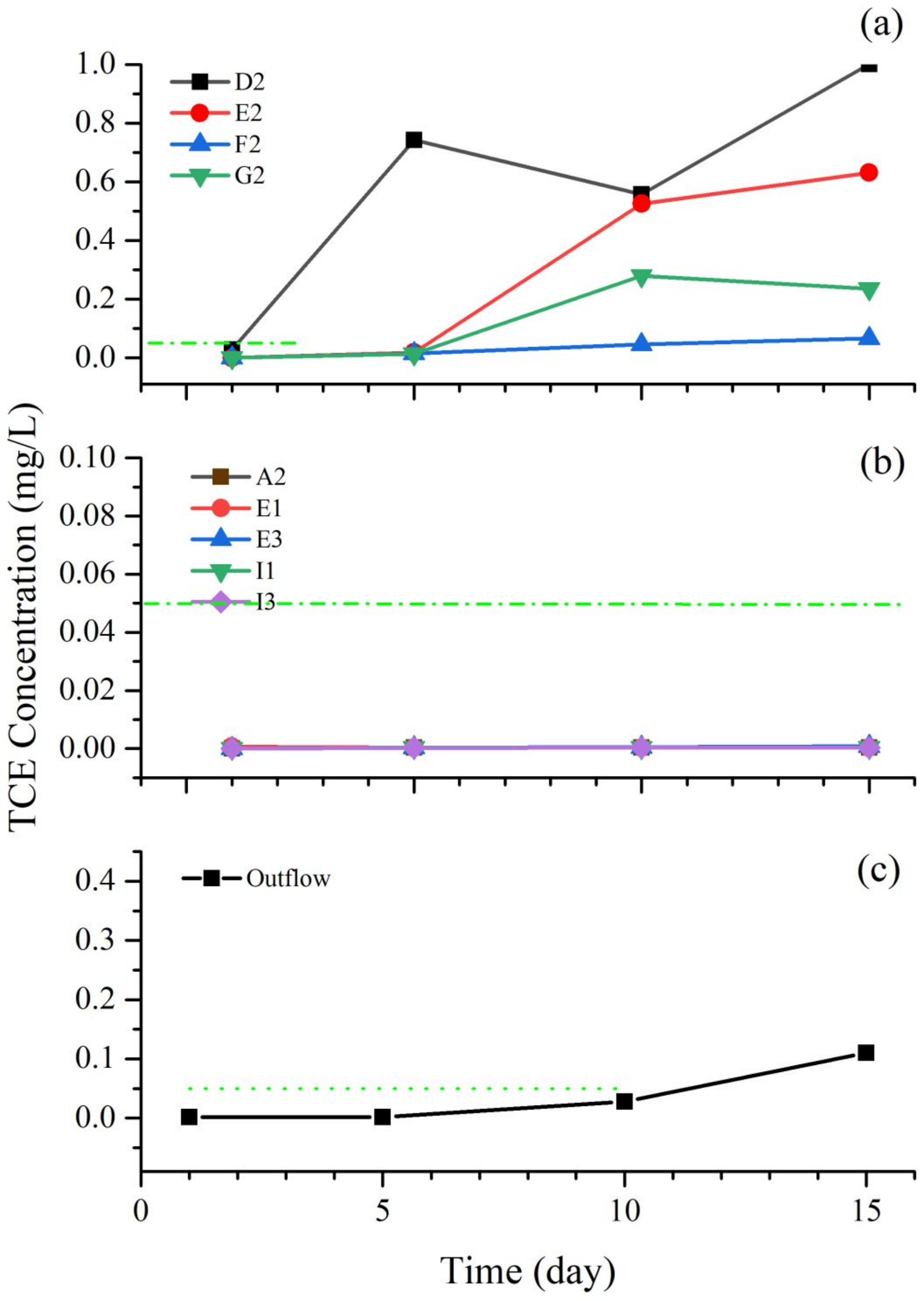

Figure S7. TCE concentration variation as a function of operation time without SPS SR-rod: (a) sampling points at LPZ, (b) sampling points at HPZ, (c) outflow

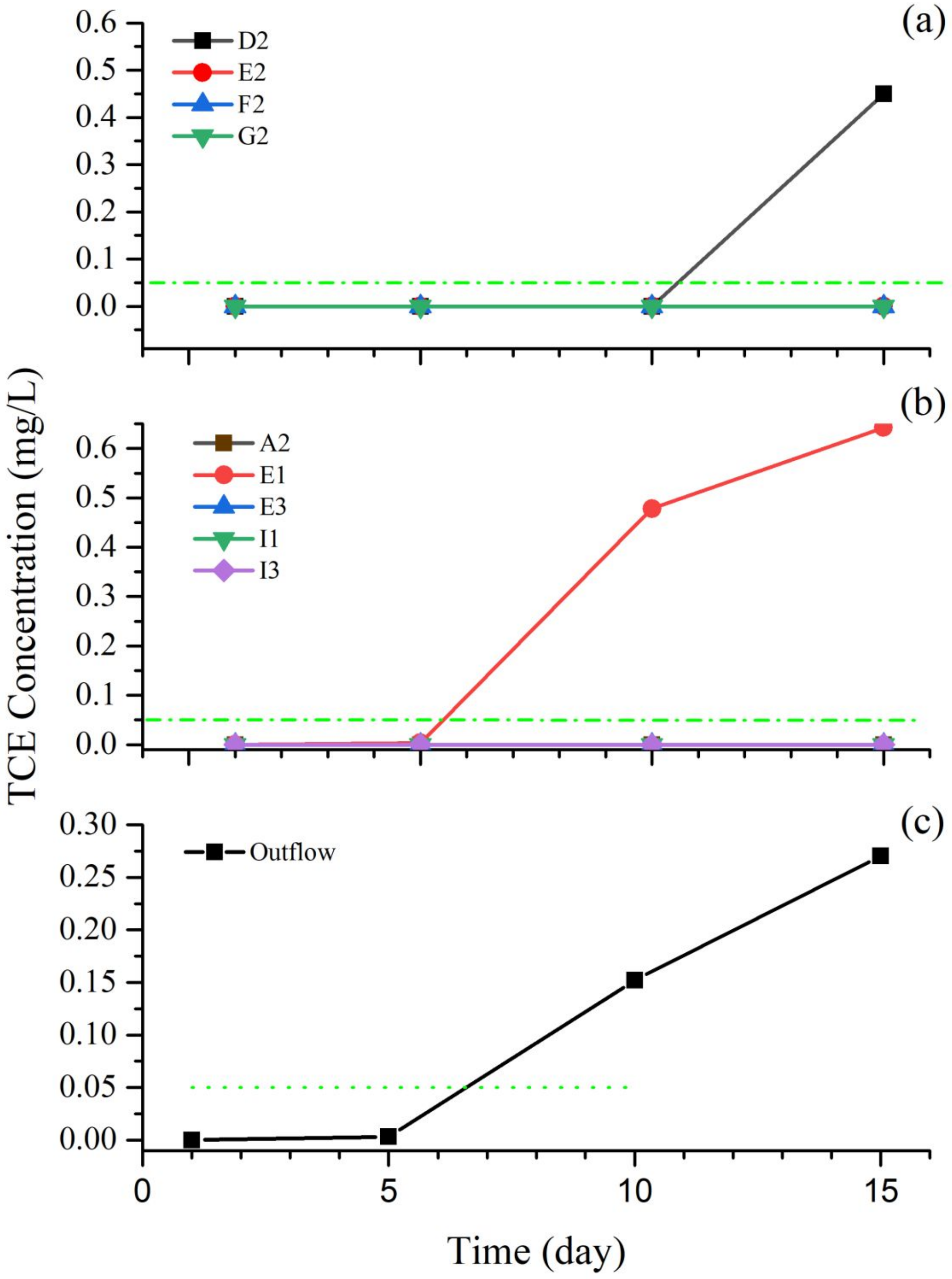

Figure S8. TCE concentration variation as a function of operation time at run WL: (a) sampling points at LPZ, (b) sampling points at HPZ, (c) outflow

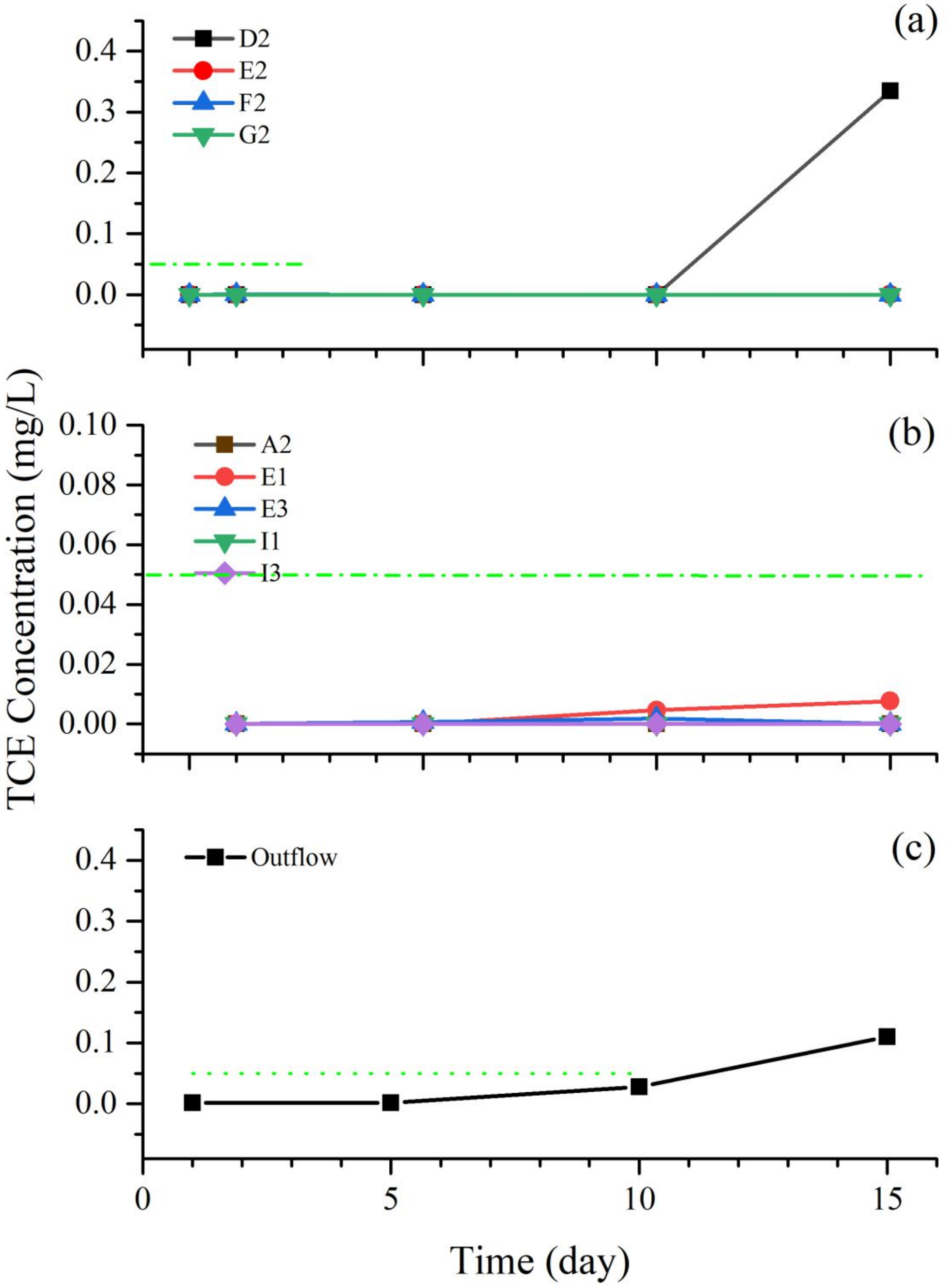

Figure S9. TCE concentration variation as a function of operation time at run AL: (a) sampling points at LPZ, (b) sampling points at HPZ, (c) outflow.

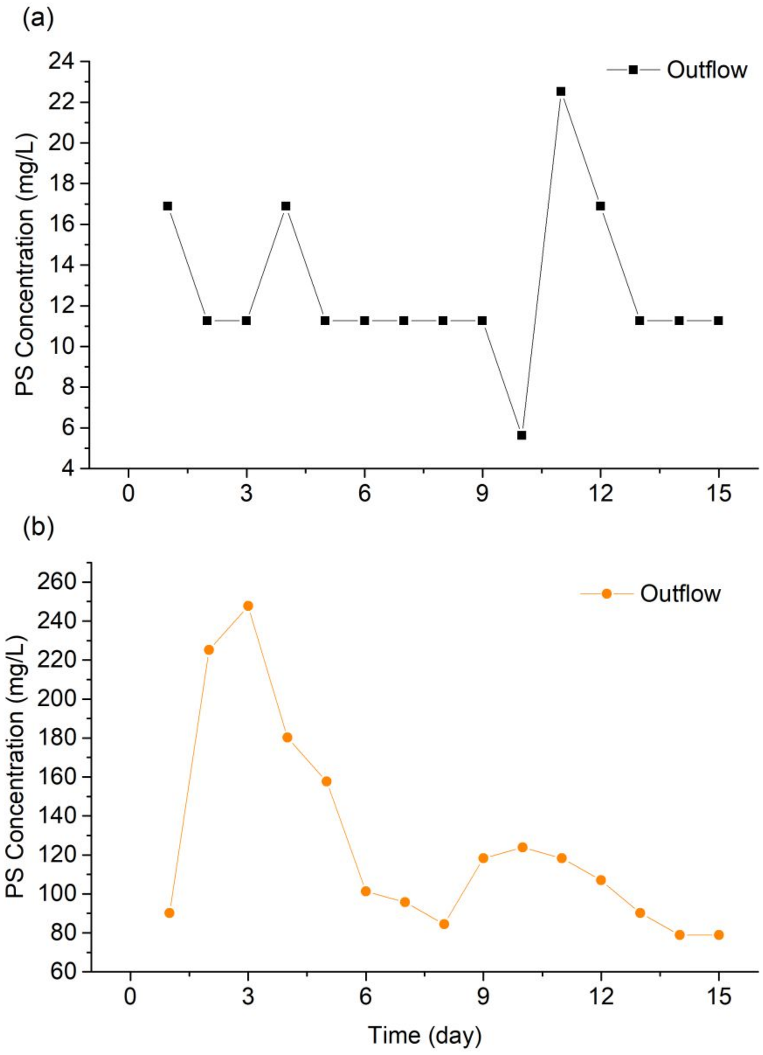

Figure S10. PS concentration in the outflow of the tank under Phase 2 experimental conditions (a) WL, and (b) AL.
